# Multiple Sentinels in a Cooperative Breeder Synchronize Rather Than Coordinate Gazing

**DOI:** 10.3390/ani13091524

**Published:** 2023-05-02

**Authors:** Guy Beauchamp, Sahas Barve

**Affiliations:** 1 Independent Researcher, Montreal, QC, Canada; 2 Archbold Biological Station, 123 Main Dr., Venus, FL 33960, USA

**Keywords:** birds, cooperative breeding, group size, sentinel behavior, synchronization, vigilance

## Abstract

**Simple Summary:**

Sentinels monitor their surroundings from vantage points for early detection of predators and rivals. The presence of multiple sentinels in a group may allow sentinels to relax their vigilance, especially if sentinels monitor different areas at the same time. We investigated sentinel behavior in groups of the Florida scrub jay (*Aphelocoma caerulescens*). Sentinels in this species turn their heads frequently to monitor different areas for potential threats. As predicted, we found that sentinels turned their heads less frequently in the presence of other sentinels. However, multiple sentinels tended to gaze in the same direction at the same time more often than predicted by chance alone. Gaze synchronization reduces the efficiency of collective detection by increasing the amount of time that some areas are not monitored by any sentinel. Despite the benefits of the presence of other sentinels, our results highlight the limits to collective detection when multiple individuals are vigilant at the same time.

**Abstract:**

Sentinels can detect predators and rivals early by monitoring their surroundings from vantage points. Multiple sentinels in a group may reduce the perceived predation risk by diluting the risk and increasing collective detection, especially if sentinels monitor different areas at the same time. We investigated sentinel behavior in groups of the Florida scrub jay (*Aphelocoma caerulescens*). Sentinels in this species turn their heads frequently to monitor different areas for threats. As predicted, we found that sentinels turned their heads less frequently in the presence of other sentinels. Multiple sentinels, however, tended to gaze in the same direction at the same time more often than predicted by chance alone. Gaze synchronization reduces the efficiency of collective detection by reducing visual coverage at any one time at the group level. Despite the benefits of the presence of other sentinels, our results highlight the limits to collective detection when multiple individuals are vigilant at the same time.

## 1. Introduction

Living in groups has long been considered an adaptation against predation [1,2]. In particular, group members can dilute risk among themselves if predators can only target one individual at a time [3]. The many eyes and ears available in a group can also allow individuals to detect predators more quickly [4]. Predator detection by a few can be passed along rapidly to others in the group, which is a process known as collective detection [5]. Models of collective detection assume that each group member monitors the surroundings independently [6,7]. Independent monitoring ensures that detection ability at the group level is directly proportional to the size of the group. In groups where individuals interrupt foraging to initiate vigilance, effective collective detection can be achieved by initiating vigilance bouts regardless of whether other group members are already vigilant or not [8].

Empirical studies on collective detection have often failed to document independent monitoring. Indeed, in many species, individuals are more likely to initiate vigilance bouts when others are already vigilant, which is a process known as synchronization [9,10,11,12,13,14,15]. Synchronization of vigilance may be costly because it increases the amount of time during which no one is vigilant at all. In addition, information about threats in the surroundings provided by several individuals that are all vigilant at the same time is more likely to be redundant thus reducing its value. Coordination of vigilance is the opposite process whereby individuals are less likely to initiate vigilance bouts when others are already vigilant [16]. Coordination, unlike synchronization, can increase the effectiveness of collective detection by decreasing the chances that no one is vigilant at all and by reducing temporal overlap in vigilance bouts at the group level. Nevertheless, coordination of vigilance is not common in animals and tends to occur in small groups where it is easier to monitor the vigilance of others, which is a prerequisite for effective coordination [10,17,18].

Collective detection could also be less effective if vigilant group members are monitoring the same areas at the same time. Indeed, if all group members only monitored one specific area during vigilance, the group would be more vulnerable to threats coming from other directions. This is likely the case in species with eyes located frontally or laterally, which makes it impossible to monitor all surrounding areas simultaneously. Such animals must therefore make frequent head turns to monitor different areas including the blind spot behind their heads [19]. To ensure effective collective detection, such animals should not only initiate vigilance bouts independently of each other, but they should also monitor different areas independently by turning their heads in different directions. This would ensure a more even monitoring of all areas at the group level.

There is limited information on collective detection in the context of acquiring information during vigilance bouts. In one study, birds in pairs synchronized their head turns suggesting that individuals paid attention to the vigilance of nearby companions [20]. However, the direction of their gaze was not examined. Other studies have examined the factors that affect the frequency of head turns but not in relation to collective detection. For instance, individuals in smaller groups or those with less experience of predators make more frequent head turns to increase visual coverage in riskier settings [21,22,23,24,25].

Here, we examined the effectiveness of collective detection during vigilance bouts in a cooperative breeder, the Florida scrub jay (*Aphelocoma caerulescens*). In this species, members of a group perch on vantage points to monitor their surroundings for possible threats such as predators and intruding neighbors [26]. Known as sentinels, these group members can detect threats more easily and pass the information along rapidly to companions foraging nearby on the ground [27]. Because their eyes are located laterally, Florida scrub jays turn their heads frequently in different directions during sentinel bouts [25], providing visual coverage of all surroundings areas including the blind spot behind their heads [28]. Occasionally, two or more group members are present during sentinel bouts. This provides a unique opportunity to examine gazing strategies in a group during long, uninterrupted vigilance bouts aimed exclusively at distant threats.

First, we aimed to determine whether sentinels benefit from the presence of other sentinels nearby. Nearby sentinels can dilute risk more effectively by reducing inter-individual distances [29]. Nearby sentinels can also monitor different areas at the same time, which improves collective detection. We predicted that the frequency of head turns in sentinels would be lower when other sentinels are present in response to the reduction in perceived predation risk. We then examined gazing strategies among multiple sentinels to gain insights into the benefits associated with collective detection. We predicted that multiple sentinels would gaze at different areas independently of one another to achieve better collective detection. The two possible alternatives to gaze independence are gaze synchronization or gaze coordination. Gaze synchronization was not expected because it implies that multiple sentinels are more likely to gaze in the same direction at the same time than predicted by chance alone thus reducing the effectiveness of collective detection. Although gaze coordination would increase collective detection benefits, it was not expected given that coordination of vigilance is not commonly reported in animals.

## 2. Materials and Methods

### 2.1. Study Site

The study was conducted at Archbold Biological Station located in south-central Florida (USA). Small scrub oaks (*Quercus* spp.) are the dominant vegetation at the station. Florida scrub jays in the area live in all-purpose territories year round and are nearly all banded and monitored regularly to evaluate group size and breeding status [30]. Juveniles in this species remain in their natal territories after the breeding season and assist breeders in territory defense and predator detection. These juveniles can transition to helper status the following breeding season and remain with the family for one or more years. Observations were carried out in the non-breeding season from late February to early March in 2022 and during the last two weeks of January in 2023. At this time of year, the number of mapped territories was about 80 and groups ranged in size from two to nine. A typical group included two breeders and two non-breeders. At least one breeder was present in each territory accompanied by juveniles, if any, from the previous breeding season and by helpers, if any, from earlier breeding seasons.

### 2.2. Sampling

Florida scrub jays were typically monitored from 7 to 11 am with a few exceptions in the late afternoon. During daily walks on different trails, one of us attempted to locate foraging groups with sentinels. Once a sentinel was detected, the observer used a video camera to record sentinel behavior. As Florida scrub jays at the station are accustomed to human presence, observations could be made at close range without obvious disturbances. A 60× zoom on the video camera also brought more distant sentinels into sharper focus, which allowed easy detection of head movements in all cases. Observations lasted for a scheduled 5 min unless the focal subject departed earlier. The observer noted how many other sentinels were present nearby and the total number of Florida scrub jays present including sentinels and foragers on the ground. Multiple sentinels in the same group were typically within a meter of one another and could all be fitted within the video shots. The identity and social status of the focal subjects were obtained later using information from the colored bands. We did not collect data during encounters between groups at territorial boundaries as sentinel behavior was rarely performed.

### 2.3. Video Analysis

We watched videos at low speed to determine the number of detectable head movements for each sentinel present in the video shots. The duration of a focal observation excluded all sequences during which the focal bird was not looking around. This included rare bouts of grooming or foraging or moments during which the focal bird was unintentionally outside the video shots.

**Figure 1:**
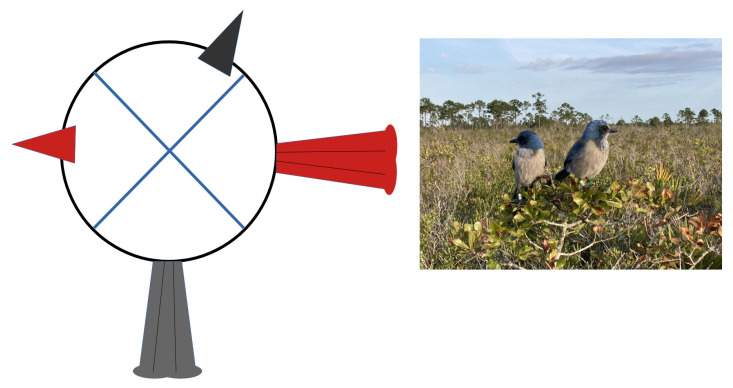
Possible orientation of the head for two sentinels to determine gaze independence. The position of the bill (filled triangle) for each sentinel was projected into one of four possible quadrants inside an imaginary circle around the head of one of the sentinels. The tail for each sentinel is shown to obtain body orientation. In this particular case, the gazes are not congruent as the bills of the two birds (shown in different colors) are positioned in different quadrants. The photograph illustrates non-congruent gazing in the field and a typical sentinel posture (photograph by GB).

For the analysis of gazing strategies among multiple sentinels, we isolated video sequences with multiple sentinels from the set of focal observations. We excluded video sequences where one of the subjects could not be seen clearly. Most cases with multiple sentinels involved only two sentinels. In rare cases where three sentinels were present at the same time, we included only two of the three possible pairs chosen randomly to maintain independence among pairs. Overall, all pairs were unique. The duration of a focal observation with multiple sentinels excluded all sequences during which either sentinel was not looking around. We adopted the following procedure to assess gaze independence. In our study, most head movements occurred on the horizontal plane. Head orientation for one of the sentinels in a pair was obtained by projecting the position of the bill onto an imaginary circle in the horizontal plane centered on the head of this bird. The bill at the zero point on this circle seen from above is aligned with the long axis of the body from head to tail and represents the orientation of the head when the bill is pointing straight ahead (Figure 1). We then determined in which of four equal segments of 90° the bill of each sentinel in the pair was positioned on the circle during gazing. Congruent gazes occurred when the two bills were in the same quadrant at the same time. Non-congruent gazes implied that the two bills were positioned in different quadrants at the same time with at least 90° between them. More congruent gazes than expected by chance alone would be compatible with gaze synchronization. Fewer congruent gazes than expected would be compatible with gaze coordination. This scoring system is admittedly coarse but realistic given the field observations. Watching the videos at slow speed, we determined the percentage of time in the focal observation during which the two bills pointed in the same general direction (within 90° of one another).

### 2.4. Statistical Analysis

In the first analysis, we wanted to determine the effect of the presence of other sentinels on the frequency of head turns for focal individuals. We used a linear mixed model to examine the association between the frequency of head turns per minute and the following fixed effects: total group size, status of the focal sentinel (breeder v. non-breeder), and type of observation (other sentinels present or not). Focal subject id was used as a random effect to account for multiple observations of the same subjects. Group id was also considered a random effect to account for multiple observations of different birds within the same group. We fitted the following model: frequency of head turns = group size + status + observation type + group size*status + group size*observation type + (1|focal subject id) + (1|group id of the focal subject).

The second analysis focused on gazing strategies among multiple sentinels. To obtain descriptive statistics for the percentage of time during which gazes were congruent among pairs of sentinels, we weighted each percentage by focal observation duration to give more weight to longer, more informative focal observations. We then computed the 95% confidence intervals around the weighted mean and determined whether it included the expected value or not.

We used the following procedure to establish the expected value. The expected percentage of time during which gazes are congruent was based on observations of lone sentinels from the 2022 field season. For those sentinels, the percentage of time that the bill was positioned in each quadrant was obtained for each subject. Based on 50 focal observations, the bill was positioned on average 50.3% of the time in the front quadrant, 19.8% and 21.3% in the two side quadrants, and 8.6% in the back quadrant. At any given time, the bill of a sentinel is thus oriented in any one of these four quadrants. The orientation of the bill over a series of discrete time steps can be viewed as a multinomial distribution with four possible, mutually exclusive outcomes (i.e., quadrants) at each time with probabilities proportional to the above four percentages. Assuming that the two sentinels of a pair monitor the surroundings independently, we can calculate the chances that the two sentinels orient their heads in the same direction using the multiplication rule of two independent random variables [31]. For instance, the chances (expressed as a percentage) that the head of each sentinel is oriented in the front quadrant is given by 50.3% times 50.3%. These chances are calculated in a similar way for the other quadrants. We limited calculations of these joint occurrences to congruent gazes, namely, when the two sentinels orient their heads in the same direction.

One difficulty in practice is that the bodies of the two sentinels may not be oriented in the same direction when their bills are pointing straight ahead. Therefore, the quadrants for each sentinel are not necessarily aligned. For instance, the front quadrants are oriented in different directions if the two sentinels face one another. To simplify calculations, we considered two extreme body orientations: parallel and antiparallel. In the parallel orientation, the bodies of the two sentinels are oriented in exactly the same direction so that all their quadrants are aligned. In the antiparallel orientation, the bodies of the two sentinels are oriented in fully opposite directions. With the antiparallel orientation, the front quadrants of each sentinel, for instance, point in the opposite direction. With these considerations in mind, we used the multiplication rule to calculate expected values for the two extreme body orientations. For congruent gazes in the parallel orientation, the expected value is given by: 50.3%*50.3% + 19.8%*19.8% + 21.3%*21.3% + 8.6%*8.6% = 34%. For the antiparallel orientation, the expected value is given by: 2*50.3%*8.6% + 2*19.8%*21.3% = 17%. Across all possible body orientations, the expected value should thus lie between 17% and 34%, which are the two extreme limits.

## 3. Results

**Figure 2:**
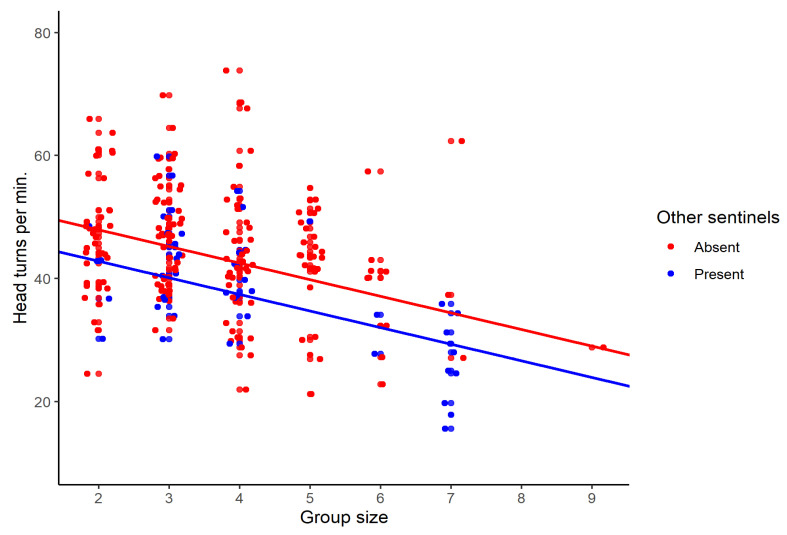
The frequency of head turns per minute for sentinels in the Florida scrub jay in groups of different sizes (n = 213). The effect of group size is shown in groups with one or more sentinels. The regression lines were obtained from a linear mixed model controlling for individual and group id.

For head turns, we obtained a total of 213 focal observations over the two non-breeding seasons, including 48 cases with multiple sentinels. Multiple sentinels typically involved two birds and occasionally three (n = 3). Focal observations lasted for a median of 95 s (range: 13 to 366 s). Groups ranged in size from two to nine with a mode of four. Sentinels included breeders (n = 149, approximately 70%) as well as non-breeders (juveniles or helpers, n = 64, approximately 30%). Sentinels turned their heads on average 43.5 times per minute ranging from 15.6 to 73.8. The head-turning rate was higher in non-breeders than in breeders (F_1,94.5_ = 14.9, *p* = 0.0002). Overall, the rate of head turning decreased with group size (β (SE) = −2.4 (0.5), F_1,170.3_ = 22.1, *p* < 0.0001; Figure 2) and was lower when other sentinels were present rather than absent (β (SE) = −5.0 (1.5), F_1,191.7_ = 10.7, *p* = 0.001; Figure 2). Using model estimates, the presence of a nearby sentinel reduced the head-turning rate more than the addition of another group member on the ground did for single sentinels (5.0 units compared to 2.4 units). There was no significant interaction between status and group size (F_1,157.7_ = 1.4, *p* = 0.24) or presence of other sentinels and group size (F_1,204.2_ = 0.84, *p* = 0.36), and both interactions were removed for the final model.

**Figure 3:**
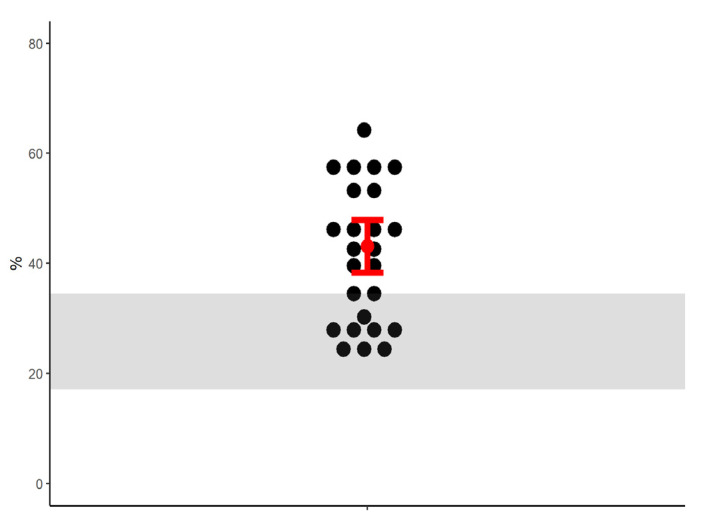
The percentage of time during which gazes were congruent between sentinels in pairs. The gray zone shows the range of percentages compatible with the null hypothesis that the two sentinels monitored the surroundings independently. Higher percentages than expected are compatible with gaze synchronization whereas lower values than expected are compatible with gaze coordination. The red lines show the 95% confidence intervals (n = 25).

We assessed gazing strategies for 25 unique pairs of sentinels. Focal observations of pairs of sentinels lasted for a median of 66 s (range: 15 to 292 s). Groups with multiple sentinels ranged in size from two to seven. We did not record any obvious interactions between multiple sentinels. Adjusting for focal observation duration, the mean percentage of time during which gazes were congruent between sentinels in a pair was 43.1% (Figure 3). The 95% confidence interval for this percentage fell above the range of expected values based on independent gazing.

## 4. Discussion

As overall group size increased, Florida scrub jays reduced the frequency of head turns during sentinel bouts. This was true for breeders and non-breeders alike although the head-turning rate was higher in non-breeders than in breeders. In addition to the overall effect of group size, the presence of other sentinels also reduced the head-turning rate. The magnitude of this effect was greater than the addition of another group member on the ground for single sentinels. Sentinels in pairs synchronized rather than coordinated gazing. Indeed, gazes in pairs of sentinels were more likely to occur in the same broad direction at the same time than expected by chance alone.

Breeding status and overall group size influenced the frequency of head turns in sentinels [25]. More experienced breeders probably have a better assessment of local predation risk than juveniles or helpers. Indeed, in many species, juveniles are more likely to give alarm calls [32], suggesting that they tend to overestimate predation risk. In larger groups, sentinels can benefit from the dilution provided by other group members foraging on the ground around them and perhaps from the ability of ground foragers to detect predators. The lower perceived predation risk in larger groups can thus translate into a lower rate of head turning during sentinel bouts.

In addition to breeding status and overall group size, the presence of other sentinels during sentinel bouts also influenced the frequency of head turns. The presence of an additional sentinel can be beneficial by increasing the dilution effect. Dilution is more effective at closer range [33,34]. The results show that the presence of a nearby sentinel had more impact on the frequency of head turns than the addition of another forager on the ground for single sentinels. Nevertheless, increased dilution alone is unlikely to explain the reduction in head-turning rate. If sentinels relied solely on the dilution effect provided by nearby sentinels, there would be no need to make head turns to detect threats. The occurrence of head turns in bouts with multiple sentinels suggests that a sentinel relied to some extent on the ability of other sentinels to detect threats. As sentinels cannot detect threats coming directly from behind, the addition of another sentinel can potentially increase the area monitored by at least one bird at any one time, which can explain to some extent why the presence of other sentinels reduced their head-turning rate.

Sentinels in pairs synchronized rather than coordinated gazing. Gaze synchronization comes at the cost of having longer periods during which no sentinel is monitoring blind spots. However, gazes were not congruent all the time, suggesting that collective detection, albeit imperfect, was not negligible. Gaze synchronization during sentinel bouts could actually bring benefits as long as congruent gazes are not too common. For instance, possible threats coming from one direction could be assessed by more than one sentinel at the same time meaning that misidentification is less likely [35]. This might be especially relevant when pairs of sentinels include breeders and younger, less experienced individuals. In addition, if predators tend to target laggards, there might be selection pressure to synchronize gaze direction [16]. This assumes that individuals detecting threats through their own eyes can flee sooner than those looking elsewhere, which can respond only after a delay to the flight responses of detectors. Future work is needed to assess these ideas.

Little empirical work has focused on gazing strategies. One study found that individuals in pairs of captive birds synchronized their head turns but it did not examine gaze direction [20]. Gaze synchronization could arise from gaze following as one individual follows the gaze of another to look at the same thing [36]. Florida scrub jays have lateral eyes and probably a wide field of view [28]. For this reason, it is not clear what exactly individuals were looking at during sentinel bouts. As we could only determine head orientation, we cannot be certain that sentinels in pairs looked at the same thing during congruent gazes. Nevertheless, from the point of view of collective detection, congruent gazes between sentinels imply that their field of view is temporarily similar and that the blind spot behind their heads is not monitored. Non-congruent gazes, by contrast, allow for a fuller visual coverage of all areas surrounding sentinels including blind spots.

Why did sentinels in the Florida scrub jay not coordinate gazing? Gaze coordination would ensure maximum collective detection. Models suggest that coordination should be more common in smaller groups [37,38], which is the case for multiple sentinels in the Florida scrub jay. However, gaze coordination requires monitoring neighbors to assess where they are looking. It is perhaps the case that the extra benefits from more efficient collective detection might not be sufficient to cover monitoring costs. A similar explanation was proposed to explain why coordination of vigilance was rare in animals that alternate between foraging and vigilance bouts [39].

Overall, the presence of other sentinels was beneficial because it decreased perceived predation risk. However, the prevalence of sentinel bouts with multiple sentinels was low in this study. Sentinels must balance the need for better vigilance from vantage points against the need to forage on the ground to accumulate resources. As a consequence, sentinel bouts for a given individual are typically not very long in the Florida scrub jay [26]. The need to resume foraging might thus set a limit on the frequency of sentinel bouts with multiple sentinels. Multiple sentinels have not been reported in Arabian babblers (*Turdoides squamiceps*) [40] but have been documented in various other species of birds such as American crows (*Corvus brachyrhynchos*) [41], white-browed sparrow-weavers (*Plocepasser mahali*) [42], jungle babblers (*Turdoides striatus*) [43], and white-winged choughs (*Corcorax melanorhamphos*) [44]. Future tests of gazing strategies can thus be performed in other species with sentinels. More broadly, tests could be performed in any situation where multiple individuals are vigilant at the same time.

## 5. Conclusions

Past research has shown that collective detection is not always effective in animals that alternate between foraging and vigilance bouts [5,45,46]. Our results show that this is also the case when considering where individuals gaze during vigilance bouts. Testing the limits of collective detection by also considering gaze direction will increase our understanding of the anti-predator function of group living in birds.

## Data Availability

The data are available from the corresponding author.
